# Optimal Perioperative Pain Management in Esophageal Surgery: An Evaluation of Paravertebral Analgesia

**DOI:** 10.1245/s10434-021-10172-1

**Published:** 2021-05-28

**Authors:** Minke L. Feenstra, Werner ten Hoope, Jeroen Hermanides, Suzanne S. Gisbertz, Markus W. Hollmann, Mark I. van Berge Henegouwen, Wietse J. Eshuis

**Affiliations:** 1 Department of Surgery, Amsterdam University Medical Centers, Location AMC, University of Amsterdam, Cancer Center Amsterdam, Amsterdam, The Netherlands; 2 Department of Anesthesiology, Amsterdam University Medical Centers, Location AMC, University of Amsterdam, Amsterdam, The Netherlands; 3 Department of Anesthesiology, Rijnstate Hospital, Arnhem, The Netherlands

## Abstract

**Background:**

For esophagectomy, thoracic epidural analgesia (TEA) is the standard of care for perioperative pain management. Although effective, TEA is associated with moderate to serious adverse events such as hypotension and neurologic complications. Paravertebral analgesia (PVA) may be a safe alternative. The authors hypothesized that TEA and PVA are similar in efficacy for pain treatment in thoracolaparoscopic Ivor Lewis esophagectomy.

**Methods:**

This retrospective cohort study compared TEA with PVA in two consecutive series of 25 thoracolaparoscopic Ivor Lewis esophagectomies. In this study, TEA consisted of continuous epidural bupivacaine and sufentanil infusion with a patient-controlled bolus function. In PVA, the catheter was inserted by the surgeon under thoracoscopic vision during surgery. Administration of PVA consisted of continuous paravertebral bupivacaine infusion after a bolus combined with patient-controlled analgesia using intravenous morphine. The primary outcome was the median highest recorded Numeric Pain Rating Scale (NRS) during the 3 days after surgery. The secondary outcomes were vasopressor consumption, fluid administration, and length of hospital stay.

**Results:**

In both groups, the median highest recorded NRS was 4 or lower during the first three postoperative days. The patients with PVA had a higher overall NRS (mean difference, 0.75; 95% confidence interval 0.49–1.44). No differences were observed in any of the other secondary outcomes.

**Conclusion:**

For the patients undergoing thoracolaparoscopic Ivor Lewis esophagectomy, TEA was superior to PVA, as measured by NRS during the first three postoperative days. However, both modes provided adequate analgesia, with a median highest recorded NRS of 4 or lower. These results could form the basis for a randomized controlled trial.

**Supplementary Information:**

The online version contains supplementary material available at 10.1245/s10434-021-10172-1.

Treatment of esophageal cancer with curative intent generally consists of neoadjuvant therapy followed by esophagectomy with gastric conduit reconstruction.^1^ Currently, most esophagectomies are performed via a minimally invasive approach.^2^ Transthoracic esophagectomy is a painful procedure due to muscular and intercostal nerve damage during surgery, and effective pain relief is important for reduction of postoperative (pulmonary) complications, including chronic postsurgical pain (CPSP), and for enhancement of patient comfort.^3^–^5^ With the introduction of minimally invasive esophagectomy, the complication rate has decreased. However, pulmonary complications still occur frequently.^6^,^7^

Currently, thoracic epidural analgesia (TEA) is recommended by the PROcedure-SPECific postoperative pain managemenT (PROSPECT) initiative for this procedure and thus reflects the standard of care in most hospitals.^8^,^9^ However, most studies assessing perioperative pain management in esophagectomy have been performed with patients undergoing open rather than minimally invasive surgery.^10^ Because of the bilateral sympathetic block, TEA is associated with more side effects, such as hypotension and urinary retention.^11^

The possible complications of TEA are dural puncture, neuralgia, epidural hematoma, and infection, at times with persistent neurologic sequaele.^12^,^13^ These adverse events occur much more frequently than previously reported.^14^,^15^ In addition to these adverse events, TEA does, at times, provide insufficient analgesia. 16,17

Paravertebral analgesia (PVA) is a safe alternative to TEA.^18^ In contrast to the blindly placed epidural catheter, PVA is performed by insertion of the paravertebral catheter into the paravertebral space intraoperatively under thoracoscopic view by the surgeon, likely increasing effective analgesia. In contrast to TEA, PVA affects the sympathetic chain unilaterally, thereby avoiding the TEA-associated side effects. During placement of the paravertebral catheter, the epidural space is spared, making development of neurologic complications (except for neurotoxicity) unlikely. Although the guidelines for PVA and TEA are similar with regard to coagulation-inhibiting drugs, PVA is likely to be safer than TEA in terms of severe bleeding complications due to its location in the paravertebral space.

The efficacy of PVA has been assessed in numerous studies of open thoracic surgery. However, only a few studies have focused on minimally invasive thoracic surgery.^19^ We hypothesized that TEA and PVA are similarly effective with regard to pain treatment in thoracolaparoscopic Ivor Lewis esophagectomy.

## Patients and Methods

### Study Design

For this single-center observational cohort feasibility study, the Institutional Medical Ethical Review Board of the Amsterdam University Medical Centers, location AMC, waived the need for informed consent. The performance and reporting of this study were in accordance with the Strengthening the Reporting of Observational Studies in Epidemiology (STROBE) guidelines.^20^

### Data Collection

A consecutive series of patients undergoing elective thoracolaparoscopic esophageal resection with two-field lymphadenectomy, gastric conduit reconstruction, and an intrathoracic anastomosis (Ivor Lewis procedure) were included from our prospectively maintained upper gastrointestinal (GI) surgery database. In December 2018, PVA was introduced as the standard of care for perioperative pain treatment of patients undergoing Ivor Lewis esophagectomy at our center. The study prospectively enrolled 25 consecutive PVA patients between December 2018 and June 2019.

Retrospectively, a historic cohort of 25 patients undergoing Ivor Lewis esophagectomies with TEA, starting January 2018, were included in the study. The only exclusion criterion was chronic opioid use longer than 3 months before surgery. Follow-up assessment was up to 30 days after surgery.

### Primary Outcome

The primary outcome was the highest recorded Numeric Pain Rating Scale (NRS) in the 3 days after surgery. The highest NRS scores at rest and during movement (movement either in bed or during coughing) were documented every shift (i.e., every 8 h) by the nursing staff.

### Secondary Outcomes

The secondary outcomes were total between-group differences in NRS, fluid administration, total fluid balance, and vasopressor consumption in the first 3 days after surgery. Finally, we evaluated the day of epidural or paravertebral catheter removal, length of stay in the post-anesthesia care unit (PACU), and length of hospital stay. Data on between-group differences in opioid consumption, intensive care unit (ICU) admission, dislocated catheters, and postoperative complications also were collected. Postoperative complications were classified according to the Clavien-Dindo classification,^21^ with Clavien Dindo scores 1–3a reflecting minor complications and Clavien Dindo scores 3b–5 indicating major complications.

All end points were retrieved from the electronic patient records except for opioid consumption in the prospective PVA cohort. In this cohort, data on opioid consumption were obtained from the patient-controlled analgesia (PCA) pumps. Opioid consumption was recalculated to morphine milligram equivalents (MMEs).

### Operative Procedure

The procedure was performed completely via minimally invasive surgery, as described in full detail elsewhere.^22^–^25^ In short, the Ivor Lewis procedure entails two phases: the laparoscopic phase and the thoracoscopic phase. In the abdominal phase, a D2 lymphadenectomy is performed, and the gastric conduit is created. In the thoracoscopic phase, a lymphadenectomy and resection of the esophagus are performed. The gastric conduit is pulled up into the thoracic cavity, and an intrathoracic end-to-side esophago-gastrostomy is created with a circular stapler 2–4 cm above the level of the carina. During the thoracic phase, a 4- to 6-cm mini-thoracotomy is performed for specimen extraction and creation of the anastomosis.

### Paravertebral and Epidural Catheter Placement and Regimens

The TEA catheter was percutaneously placed at an intervertebral level between Th5 and Th8 using the loss-of-resistance technique and then introduced 3–6 cm into the epidural space before the induction of general anesthesia. During surgery, a mixture of bupivacaine 0.125% and sufentanil 0.5 μg/mL was infused via the epidural catheter at 6 to 10 ml/h. Further intraoperative pain management was left to the discretion of the treating anesthesiologist.

Postoperatively a patient-controlled bolus function (PCEA) was initiated on top of the continuous infusion. The patients could bolus 1 ml every 5 min, to a maximum of 30 ml every 4 h.

**Fig. 1:**
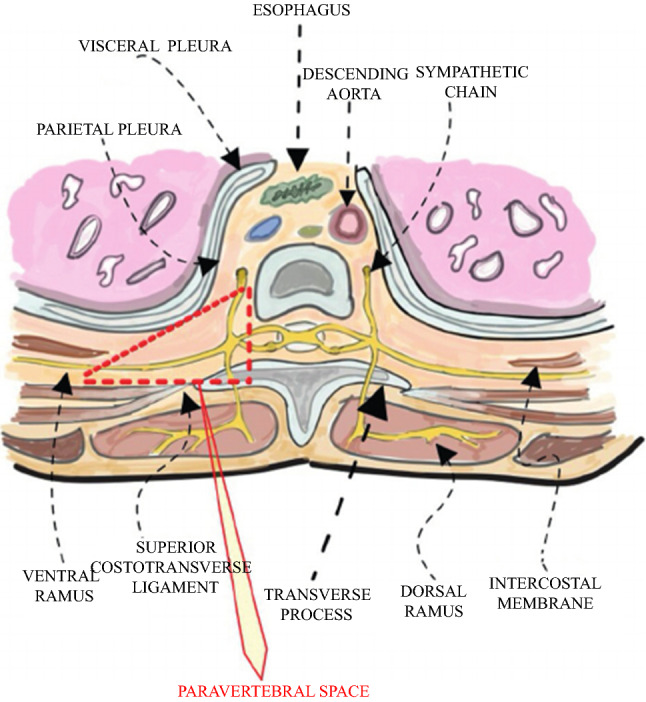
The paravertebral space (thoracal paravertebral block for breast surgery; Beyaz et al. *Dicle Med J.* 2012)

The paravertebral catheter was placed by the surgeon under thoracoscopic vision in the paravertebral space lateral to level Th4-Th5 on the right side and ipsilateral to the mini-thoracotomy immediately after placement of the patient in the prone position, and the trocars were placed in the thorax to start the thoracic phase of the surgery. First, a bolus of 20 ml bupivacaine 0.125% was administered. Postoperatively, a continuous infusion of bupivacaine 0.125% at 8–12 mL/h was initiated (Fig. 1). Further intraoperative pain management was left to the discretion of the treating anesthesiologist. Postoperatively, the patients in the PVA group were also provided with an intravenous PCA pump. Either morphine or buprenorphine, depending on the patient’s glomerular filtration rate, was given. The patients could bolus 1 ml (1 mg morphine or 30 μg buprenorphine) every 5 min, to a maximum of 30 ml every 4 h.

After surgery, all the patients without contraindications received paracetamol (4 × 1 g) and metamizole (4 × 1 g). In case of inadequate analgesia, escape medication was provided. In both PVA and TEA, the catheter was routinely removed on day 3 after surgery. Analgesia via the catheter was stopped a few hours before its removal to check whether pain treatment was still sufficient.

### Sample Size Calculation

We decided to include 25 patients in both groups. This enabled us to detect a clinically relevant NRS difference of 2 with an alpha of 0.05 and a power of 80%, assuming a standard deviation of 2.5 (based on Kingma et al.^16^). Sample size calculation was performed with NQuery (version 8.5.1.0; Statistical Solutions Ltd. Cork, Ireland).

### Statistical Analysis

All other analyses were executed using SPSS version 25 (IBM Corp., Armonk, NY, USA). Data are expressed as mean ± standard deviation for normally distributed continuous variables, as median and interquartile range (IQR) for non-normally distributed variables, and as proportions for binary variables.

Continuous unpaired data were compared using the Mann-Whitney *U* test or the independent-samples *t* test, as appropriate. To compare categorical data, the chi-square or Fisher’s exact test was used, as appropriate. Missing values were not imputed, and complete case analysis was performed.

For repeated measurements analyses, a linear mixed model was used. Several covariance matrices were tested for the best fit using restricted maximum likelihood. The covariance matrices tested were auto regressive 1, diagonal, Toeplitz, and unstructured matrices. After determination of the best covariance matrix, parameter estimates were calculated using maximum likelihood. Two-tailed probabilities were calculated, with values lower than 0.05 considered statistically significant. To correct for multiple testing in the results, the Benjamini Hochberg method was applied.^26^

## Results

**Fig. 2:**
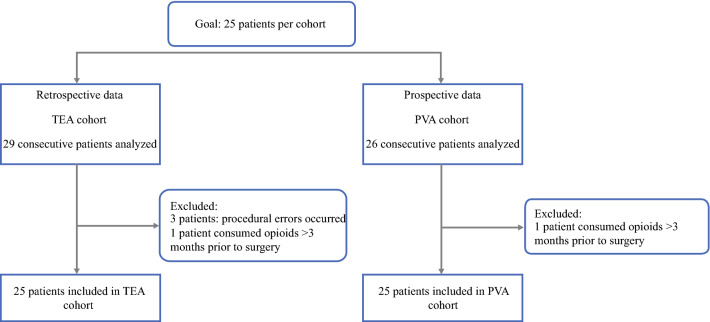
Flowchart of patient inclusion.

From December 2018 to June 2019, 25 consecutive patients were prospectively included in the PVA group (Fig. 2). Retrospectively, 25 consecutive patients with TEA were analyzed, from January 2018 to August 2018. Four patients with TEA were excluded from the study, including one patient who had consumed additional opioids without knowledge of the physicians, one patient who had continuous epidural analgesia without bolus function, one patient who had chronic opioid use, and one patient who had continuous epidural analgesia with only a local anesthetic. One patient with PVA was excluded due to chronic opioid use. All catheters were placed successfully.

The baseline characteristics of the patients are shown in Table 1. On the average, six NRS measurements from a total of 22 NRS scores per patient at rest and during movement were missing. Most of these missing NRS scores were the NRS scores at night. The data on opioid consumption were missing for 6 TEA patients and 14 PVA patients. The data on vasopressor consumption was missing for 3 TEA patients and 4 PVA patients.

**Table 1:** Baseline characteristics

	Epidural analgesia (*n* = 25) *n* (%)	Paravertebral analgesia (*n* = 25) *n* (%)
Mean age (years)	63.5 ± 8.0	66.2 ± 7.9
Mean BMI (kg/m^2^)	25.6 ± 3.4	27.2 ± 4.2
Gender (female)	4 (16.0)	4 (16.0)
ASA		
1	3 (12.0)	1 (4.0)
2	14 (56.0)	15 (60.0)
3	8 (32.0)	9 (36.0)
Neoadjuvant therapy		
Chemotherapy	2 (8.0)	3 (12.0)
Chemoradiotherapy	23 (92.0)	20 (80.0)
No neoadjuvant therapy	0	2 (8.0)
Previous abdominal surgery	3 (12.0)	7 (28.0)
Previous thoracic surgery	0	1 (4.0)

### Primary and Secondary Outcomes

**Fig. 3:**
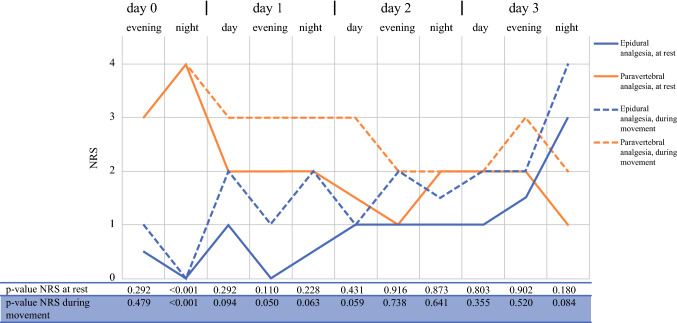
Median NRS with epidural or paravertebral analgesia. The *y*-axis shows the NRS, and the *x*-axis shows the time expressed in days and shifts (e.g., the first shift was the evening shift immediately after surgery). The *p* values are corrected for multiple testing with the de Benjamini–Hochberg method

The median highest NRS scores recorded per 8-h shift are shown in Fig. 3. The first night after surgery, the patients in the PVA cohort experienced more pain than the patients in the TEA cohort at rest and during movement (median NRS, 4 vs 0; *p* < 0.001). However, in both groups, the median NRS score was moderate (≤ 4). During the evening shift on day 1 after surgery, the PVA cohort had a higher NRS score during movement (3 vs 1; *p* = 0.050). On days 2 and 3 the NRS scores did not differ significantly between the two groups.

The overall NRS at rest showed significantly higher scores in the PVA cohort than in the TEA cohort (mean difference, 0.75; 95% confidence interval [CI] 0.49–1.44), as did the NRS during movement (mean difference, 0.97; 95% CI 0.19–1.74).

No significant difference in fluid administration, fluid balance, or vasopressor consumption was observed, but all three were higher in the TEA cohort (respective mean differences of 72.2 mL [95% CI − 193.6 to 338.1 mL], 183.1 mL [95% CI − 109.3 to 475.6 mL], and 134.8 μg [95% CI − 91.1 to 360.8 μg]). On day 2, three patients needed vasopressors: two patients in the TEA cohort and one patient in the PVA cohort. The number of patients requiring vasopressors did not differ significantly between the two cohorts on day 0, 1, 2, or 3 after surgery. Only norepinephrine was used as a vasopressor. One patient with TEA had severe hypotension, which resulted in early removal of the epidural catheter.

The TEA cohort received more opioids than the PVA cohort (mean difference, 68.7 MMEs; 95% CI 47.8–89.6 MMEs). No patient in the TEA cohort required escape medication, whereas two patients in the PVA cohort required escape medication in the form of clonidine at standard times, with a maximum of 300 μg of clonidine per day. No patients experienced local anesthesia or opioid intoxications or ileus. Four catheters were dislocated in the PVA cohort compared with three catheters in the TEA cohort. Postoperative complications, ICU admission, and length of hospital or PACU stay did not differ between the two cohorts (Table 2).

**Table 2:** Secondary end points, other

	Epidural analgesia (*n* = 25) *n* (%)	Paravertebral analgesia (*n* = 25) *n* (%)	*p* value^a^
Median PACU stay: hours (IQR)	17.0 (15.6–18.9)	17.0 (16.4–17.7)	> 0.99
Median hospital stay: days (IQR)	12.0 (9.0–15.5)	12.0 (9.0–20.8)	0.91
Median postoperative day of catheter removal	3.0 (3.0–4.0)	3.0 (3.0–4.0)	0.91
Failed catheter	3 (12.0)	4 (16.0)	0.91
Admission ICU	2 (8.0)	7 (28.0)	0.59
Pneumonia	1 (4.0)	3 (12.0)	0.79
Anastomotic leak	3 (12.0)	4 (16.0)	0.91
No complications	12 (48.0)	8 (32.0)	
Minor complications^b^	8 (32.0)	8 (32.0)	
Major complications^c^	5 (20.0)	9 (36.0)	0.68

^a^Corrected for multiple testing with the Benjamini–Hochberg method

^b^Clavien Dindo classs 1–3a

^c^Clavien Dindo classes 3b–5

## Discussion

This cohort study focused on the efficacy of TEA compared with PVA for patients undergoing thoracolaparoscopic esophagectomy with an intrathoracic anastomosis. Overall, TEA was superior to PVA with regard to the median highest recorded NRS scores within the first three postoperative days. This was mostly due to the first night after surgery, during which the patients with TEA had lower NRS scores than the patients with PVA. However, the pain in the PVA cohort was moderate (median NRS score, 4). In addition, the TEA cohort received more opioids than the PVA cohort due to the continuous sufentanil administration in the TEA group. No difference in fluid administration, fluid balance, or vasopressor consumption was observed. The incidence of postoperative complications, ICU admission, and length of hospital and PACU stay did not differ between the two cohorts.

This is the first study to compare TEA with PVA in minimally invasive esophagectomy. In a Cochrane Review of TEA and PVA in open thoracic surgery, Yeung et al.^19^ concluded that TEA and PVA resulted in similar postoperative pain levels. Van den Berg et al.^27^ evaluated PVA in minimally invasive Ivor Lewis esophagectomy without a comparator and considered PVA to be effective and safe. A possible explanation for lower NRS scores the first night after surgery for the patients with TEA compared with PVA is the continuous infusion of epidural opioids in the TEA group and inadequate use of PCA morphine in the PVA group. This is the standard of care, but inevitably increases opioid consumption in the TEA group. Also, the patients with PVA might have required morphine after already considerable pain. Either the patients awoke from pain and requested more opioids too late or they were satisfied with their level of analgesia and did not ask for more opioids. The latter would suggest overtreatment with opioids in the TEA group.

No side effects or adverse events due to opioid consumption occurred in this study. When TEA is used, the sensory block is more extended, with most (if not all) the thoracic and abdominal surgical wounds anesthetized. This also could possibly explain the lower NRS scores for the TEA patients. Due to the retrospective nature of this study, the adequacy of locoregional sensory block could not be assessed. Another possible explanation is the learning curve for insertion of the paravertebral catheter. Because the technique of inserting the paravertebral catheter was implemented 2 months before the start of the study, the influence of a learning curve effect is unlikely. However, it cannot be ruled out completely.

Because PVA leads to a unilateral block, fewer side effects, such as hypotension, than with TEA can be expected. Several studies have shown that PVA reduces postoperative hypotension compared with TEA.^19^,^28^,^29^ We could not confirm these findings in our cohort; nor did we find a difference in fluid administration or vasopressor consumption. Apparently, our sample was too small for detection of a difference in these outcomes.

A major advantage of PVA, albeit not investigated in this study, is the lack of any unpleasant experience during epidural catheter placement, which is commonly performed for the awake patient. However, due to the retrospective nature of this study, patient-reported outcomes could not be evaluated. Furthermore, the time frame of the observed cohorts may have influenced the results. The TEA cohort underwent surgery from January to August of 2018, whereas the PVA cohort had surgery from December 2018 to June 2019. Although the interval was shorter than a year, changed habits in clinical practice still could have occurred, which could have resulted in bias. In addition, missing data for several variables, which is inherent to the retrospective collection of data, might have biased our results.

Because of the limited information on opioid consumption in the PVA group, these results should be interpreted with caution. However, due to the continuous infusion of opioids in the TEA group, it still is most likely that the TEA group consumed more opioids. Also, at night, the NRS score often was not recorded. Most likely, NRS scores were not recorded because the patients were asleep, and lower NRS scores may thus have been missed, potentially skewing the data.

In this study, pain was evaluated by NRS scores. Although NRS scores are widely implemented as pain measurements, they also are limited because they only partially reflect patient satisfaction. A more complete patient-reported outcome is required. The QoR-40, a validated patient-reported outcome designed in Australia by Myles et al.,^30^ evaluates the quality of overall recovery after anesthesia. It provides a patient-reported outcome evaluating five domains: emotional state, physical comfort, psychological support, physical independence, and pain. The questionnaire was validated in 2000, and since then has been translated and implemented in many different countries. Future studies should focus more on patient-reported outcomes such as the QoR-40.

In conclusion, the study showed that TEA was superior to PVA regarding NRS scores for patients undergoing thoracolaparoscopic Ivor Lewis esophagectomy, although both methods provided adequate analgesia with a median NRS of 4 or lower. This was the first study to compare PVA and TEA in thoracolaparoscopic Ivor Lewis esophagectomies, and the results should encourage adequately powered randomized controlled trials assessing the effectiveness of PVA for this procedure, such as the PEPMEN trial with its study protocol published recently using patient-reported outcomes.^31^

## Supplementary Information

Below is the link to the electronic supplementary material.

Supplementary file1 (DOCX 29 kb)
